# Non-parametric mixture modeling of cognitive psychological data: A new method to disentangle hidden strategies

**DOI:** 10.3758/s13428-022-01837-0

**Published:** 2022-10-11

**Authors:** Kim Archambeau, Joaquina Couto, Leendert Van Maanen

**Affiliations:** 1 Department of Psychology, University of Amsterdam, Postbus 15906, 1001 Amsterdam, NK Netherlands; 2 Center for Research in Cognition and Neurosciences, Université Libre de Bruxelles, Brussels, Belgium; 3 Department of Experimental Psychology, Utrecht University, Heidelberglaan 1 – 3584, Utrecht, CS Netherlands

**Keywords:** Strategy, Mixture modeling, Hidden Markov model, Non-parametric model

## Abstract

In a wide variety of cognitive domains, participants have access to several alternative strategies to perform a particular task and, on each trial, one specific strategy is selected and executed. Determining how many strategies are used by a participant as well as their identification at a trial level is a challenging problem for researchers. In the current paper, we propose a new method – the non-parametric mixture model – to efficiently disentangle hidden strategies in cognitive psychological data, based on observed response times. The developed method derived from standard hidden Markov modeling. Importantly, we used a model-free approach where a particular shape of a response time distribution does not need to be assumed. This has the considerable advantage of avoiding potentially unreliable results when an inappropriate response time distribution is assumed. Through three simulation studies and two applications to real data, we repeatedly demonstrated that the non-parametric mixture model is able to reliably recover hidden strategies present in the data as well as to accurately estimate the number of concurrent strategies. The results also showed that this new method is more efficient than a standard parametric approach. The non-parametric mixture model is therefore a useful statistical tool for strategy identification that can be applied in many areas of cognitive psychology. To this end, practical guidelines are provided for researchers wishing to apply the non-parametric mixture models on their own data set.

## Introduction

When people are faced with making a decision or solving a problem, often a number of alternative strategies can be applied. Using different strategies to perform a particular task has indeed been observed in a wide variety of cognitive domains such as arithmetic problem solving (e.g., Campbell & Xue, 2001; Siegler & Lemaire 1997), economic decision-making (Couto, Van Maanen, & Lebreton, 2020 ; Lopez-Persem, Domenech, & Pessiglione, 2016; Payne, Bettman, Schakde, Schwarz, & Gregory, 1999), lexical production (Kail, Lemaire, & Lecacheur, 2012; Sprenger & Van Rijn, 2013), categorization (Palatano, Smith, Jonides, & Koeppe, 2001; Smith, Patalano, & Jonides, 1998) or memory (e.g., Donkin, Nosofksy, Gold, & Shiffrin, 2013; Dunlosky & Hertzog, 2001; Touron & Hertzog, 2009). Taking as an example the arithmetic field, solving simple arithmetic problems (e.g.,“7 x 6”) could be achieved by either retrieving the solution from long-term memory (e.g., Archambeau, De Visscher, Noël, & Gevers, 2019) or by using several cognitive processing steps (e.g., 7 x 6 = (6 x 6) + 6 = 42; LeFevre et al., 1996).

A difficult and as of yet unsolved problem pertains to the identification of such different strategies. A participant could select one specific strategy on a particular trial and then alternate for another strategy on the next trial. As a consequence, the observed behavior over trials results from a mixture of several strategies. However, it is typically unknown how many strategies are used by a participant to perform a task and which strategy is selected on a given trial.

In order to assess cognitive strategies, researchers frequently administered self-report instruments where participants are asked to describe the strategy used to solve the task (e.g., Campbell & Xue, 2001; LeFevre et al., 1996; Touron & Hertzog, 2009). However, self-report instruments are not ideal because asking for a report can influence the performance or change the selected strategy (e.g., Fox, Ericsson, & Best, 2011). Additionally, participants are not always aware of the strategy they followed (e.g., Crutcher, 1994; Ericsson & Simon, 1984; Kirk & Ashcraft, 2001; Veenman, 2011).

A solution to the problems associated with self-report instruments is to rely on systematic differences in behavior that can be associated with different cognitive strategies. In the arithmetic example introduced before, it was observed that the multistep solution strategy requires more processing time than the long-term memory-based solution strategy, so faster responses are more likely to be the result of a single memory process rather than a sequence of cognitive processing steps, as required for a multistep solution (e.g., Campbell & Xue, 2001; LeFevre et al., 1996).

A statistical method that quantifies differences in behavior associated with different cognitive strategies is called a hidden Markov model (HMM). A HMM assumes that the observed data are generated by multiple hidden states that produce a specific pattern of observable behaviors (e.g., Visser, 2011; Zucchini, MacDonald, & Langrock, 2017). For most applications in cognitive psychology, these observable behaviors are response times (RT) and accuracy data. Another core property of HMM is the transition dynamics between the hidden states. These transition dynamics are represented by the transition probabilities of a Markov chain, which are the probabilities of remaining in the same state and of switching to another state (Visser, Raijmakers, & van der Maas, 2009). Under the assumption that each cognitive strategy is reflected by a different set of behaviors, they can be represented by different hidden states in HMM (for an example, see Dutilh, Wagenmakers, Visser, & van der Maas, 2011)^1^. In short, HMM allows researchers to learn about latent strategies present in the data: their number, their behavioral characteristics, their transition dynamics, and their identification at a trial level. For an extensive explanation about HHM, we refer the interested reader to excellent tutorials already present in the literature (e.g., Rabiner, 1989; Visser 2011; Visser et al., 2009; Zucchini et al., 2017).

For applying HMM, the RT distribution within each state has to be specified and often the assumption of a normal distribution is made^2^ (e.g., Dutilh, 2011; Mair, 2018; Visser, 2011; Visser et al., 2009 but for an exception see Kucharsky, Tran, Veldkamp, Raijmakers, & Visser,^2021^). However, it is typically observed that RT in cognitive psychological data are not normally distributed (e.g., Anders, Alario, & Van Maanen, 2016; Matzke & Wagenmakers, 2009; Wagenmakers & Brown, 2007). Crucially, Molenaar et al. (2018) demonstrated that a violation in the assumed RT base distribution could lead to unreliable results. That is, the incorrect number of states may be inferred and strategy categorization may be biased (Molenaar, Bolsinova, & Vermunt, 2018). One obvious solution to solve this is to use a more suitable RT distribution for the latent states. However, this solution is not that feasible since, as previously mentioned, the states are not directly observable. Therefore, determining the true base distribution is extremely challenging (for a similar proposal, see Molenaar et al., 2018). In the current paper, we present another – model-free – solution that remains agnostic about the particular shape of the RT distribution. More specifically, we categorized RT into ranges of equal length and we model this according to a multinomial frequency distribution. This means that the HMM only uses information about what range of RT occurs more often than what other range, but ignores the actual RT. This is similar to the semi-parametric HMM developed by Molenaar et al., (2018; see also Molenaar, Rósza, & Bolsinova, 2019) but simplified in the sense that even the ordinal information with respect to the RT ranges is ignored. Given that our approach uses less information than the *semi-parametric* approach of Molenaar et al., (2018), we termed this method *non-parametric mixture model*, to be consistent with their terminology. The current method focuses then on one of the most measured variables used in cognitive psychology: RT. Note that other approaches, which are not based on HMM and on RT, have also been developed to efficiently disentangle cognitive strategies. The current paper does not address those but see for instance, Lee, Gluck, & Walsh, (2019), Lee & Gluck, (2020) or Steingroever, Jepma, Lee, Jansen, & Huizenga, (2019) for more details.

The goal of the current paper is to investigate whether the non-parametric mixture model is a suitable method for disentangling hidden strategies in cognitive psychological data. More precisely, we aim at demonstrating that the non-parametric mixture model is able to reliably recover hidden states present in the data as well as to accurately estimate the number of concurrent strategies. Additionally, we compared model performance of the non-parametric mixture approach to the semi-parametric and parametric mixture approaches. It should be noted that semi-parametric mixture model was only implemented in the psychometric field (Molenaar et al., 2018; Molenaar et al., 2019). Therefore, its efficiency with typical cognitive psychological data needs to be validated. To achieve this, the non-parametric mixture model was tested by the way of two simulation studies and by its application on real data from two different domains of cognitive psychology. More precisely, in Simulation 1 and 2, the efficiency of the method was first investigated on data where RT were simulated according a shifted Wald distribution (SWD) and accuracy according to a binomial distribution. SWD was chosen because it typically mimics most of observed RT data in cognitive psychology (Anders et al., 2016; Heathcote, 2004). In Simulation 3, the non-parametric mixture model was applied on accuracy and RT data generated using a cognitive model. After these three simulations studies, the method was tested on real data sets to demonstrate its applicability in different fields of cognitive psychology. First, we applied the method in the perceptual decision making field (Experiment 1) and then in the economic decision making field (Experiment 2).

## Simulation studies

In order to validate the non-parametric mixture method, three simulation studies were run. Two simulations involved RT drawn from a SWD, and accuracy from a binomial distribution. More precisely, the goal of the first simulation was to investigate which kind of mixture data could induce a poor model performance. The aim of the second simulation was to demonstrate that the proposed method is also efficient in recovering more than two hidden states. Finally, the third simulation involved RT and errors generated by a cognitive model of binary choices, the diffusion decision model (DDM).

### Simulation 1: Which mixture data induces a poor model performance?

In Simulation 1, the non-parametric mixture method was applied to behavioral data where the RT of both states are generated according to a SWD. SWD was chosen because it has a right-skewed shape that typically mimics most RT distribution of cognitive psychological data (e.g., Anders et al., 2016; Heathcote, 2004). Accuracy for both states were sampled from two binomial distributions. In this first simulation, we further investigated which kind of mixture data was susceptible to exhibit poorer model performance by systematically varying parameter features of the generated data (e.g., mean RT difference between both mixture, skewness of the distribution, number of trials).

#### Method

##### 1.1.1.1. Baseline parameters

RT data for both states were sampled from two SWDs. Given the location, shape, and shift parameters that determine the SWD (*μ*, *λ*, *τ*, respectively), the expected M, SD, and skewness (SK) of RT are:

$$M=\mu +\tau SD=\sqrt{\frac{\mu^3}{\lambda }} SK=3\sqrt{\frac{\mu }{\lambda }}$$

We used these mappings to compute the parameters for a particular combination of M, SD, and SK (see Supplementary Information^3^). The first state was generated with the following SWD parameters: *M*_*1*_ = 600 ms, *SD*_*1*_ = 100, *SK*_1_ = 1.5. This state was also associated with an accuracy of 70%. The second state was associated with an accuracy of 90% and with the following SWD parameters : *M*_*2*_ = 900 ms, *SD*_*2*_ = 150, *SK*_2_ = 1.75. In short, the first state corresponds to relatively fast and more error-prone behavioral responses and the second state corresponds to relatively slow and more accurate behavioral responses. These arbitrary values seem a reasonable representation of a real cognitive psychological data set involving two different strategies (Luce, 1986). We generated 500 observations for each of the 100 simulated participants. The mixture proportion was equal between both states. That is, both states are equally likely and have the same number of observations (i.e., 250). The transition parameters of the Markov chain were set to 0.8 and 0.2 for both states, meaning that the probability of remaining in the same state is 0.8 and the probability of switching to the other state is 0.2. SWD and Markov chain were respectively generated using the “SuppDists” package (Wheeler, 2008) and the “markovchain” package (Spedicato et al., 2016) for R program (R Development Core Team, 2016).

##### 1.1.1.2. Model specification

The HMMs were fitted to individual data using the “depmixS4” R package (Visser & Speekenbrink, 2010). The HMM specification of all three mixture models (i.e., parametric, semi-parametric, and non-parametric) included multivariate data (RT and accuracy). For the three mixture models, the accuracy variable was modeled as a multinomial distribution with an identity link function. For the parametric mixture model, RT was modeled assuming a Gaussian distribution. This is the default and usually chosen distribution (e.g., Dutilh et al., 2011; Visser 2011; Visser et al., 2009). Because, as previously mentioned, the states are hidden, there is no way to infer their specific distributions beforehand. For the semi-parametric and non-parametric mixture models, individual RT data were first discretized into bins of equal length (Molenaar et al., 2018). The number of bins was set to 20 for both mixture models. For the non-parametric mixture model, RT bins variable was modeled as a multinomial distribution with a logit link function. For the semi-parametric mixture model, the RT bins variable was modeled as an ordinal distribution with a logistic link function. Because the ordinal distribution was not present by default in the “depmixS4” R package (Visser & Speekenbrink, 2010), we implemented the ordinal fitting using the *polr* function of the MASS R package (Ripley et al., 2013). All models assumed two hidden states. Parameters of HMMs were estimated with the expectation-maximization algorithm. For each simulated participant and model, the fitting procedure was repeated 20 times and the iteration with the lowest BIC (Schwarz, 1978) was selected.

##### 1.1.1.3. Parameter manipulation

In order to investigate the impact of different features of the data on the efficiency of identifying hidden states, a series of simulations were run. For all features (except mixture proportion), we used two levels (low and high) of manipulation. The *low* level refers to a decrease in the manipulated feature whereas the *high* level refers to an increase in the manipulated feature. When one feature was manipulated, the others were kept constant to their baseline value. Table 1 summarizes the manipulated features and their corresponding values for baseline, low and high levels. As can be seen, some manipulated features concern the SWD: RT difference, standard deviation and skewness. For the RT difference manipulation, the mean difference between both states was modified by decreasing/increasing the mean RT of the slowest state. The standard deviation and skewness manipulations changed both state distributions by multiplying the baseline value with 2/3 and 4/3 for low and high levels, respectively. In a similar way of RT manipulation, the accuracy difference between both states was also examined by decreasing/increasing the mean accuracy of the less accurate state. One manipulation investigated the impact of the sample size by varying the number of trials. One manipulation explored model stability (i.e., the probability of remaining in the same state) by varying transition parameters of the Markov chain. Because the number of bins is arbitrary set for semi-parametric and non-parametric mixture models, we explored how this feature is important and could influence model accuracy. Finally, the mixture proportion manipulation tested the influence of unequal proportion for the hidden states (i.e., one state is more likely and then associated with more trials than the other).

**Table 1:** Manipulated features of the data for Simulation 1

	Low	Baseline	High
Reaction times difference	150ms	*300ms*	600ms
Accuracy difference	10%	*20%*	40%
Skewness	SK*2/3	SK*1	SK*4/3
Standard Deviation	SD*2/3	SD*1	SD*4/3
Model Stability	0.65-0.35	*0.8-0.2*	0.95-0.05
Number of trials	126	*500*	2000
Number of bins	5	*20*	35
Mixture Proportion	Unequal (proportion >.65)	Equal	Unequal (proportion >.65)

Values for the baseline as well as the low and high levels of manipulation for each manipulated features of the data. For each manipulated feature, the others are kept fixed to their baseline value (*in italics*)

##### 1.1.1.4. Model selection

In a model selection procedure, we investigated how many states provides the best fit to the data. Therefore, four different models varying the number of states from 1 (i.e., assuming no mixture) to 4 were fitted to the simulated data with the baseline values. All other model specifications were kept constant. We examined model selection according to two statistics for goodness-of-fit : BIC (Schwarz, 1978) and AIC (Akaike, 1974). While BIC is commonly used in mixture data and HMM in particular (e.g., Visser, 2011), AIC was also provided because it is commonly used in mathematical psychology. BIC and AIC tend to complement each other as, typically, AIC favors more complex models, whereas BIC favors simpler models (e.g., Van Maanen et al. 2016). BIC and AIC were computed for each simulated participant and each model. The model with the lowest BIC and AIC for the majority of simulated participants is selected as the best model. All these steps were performed for each mixture model.

#### Results

**Fig. 1:**
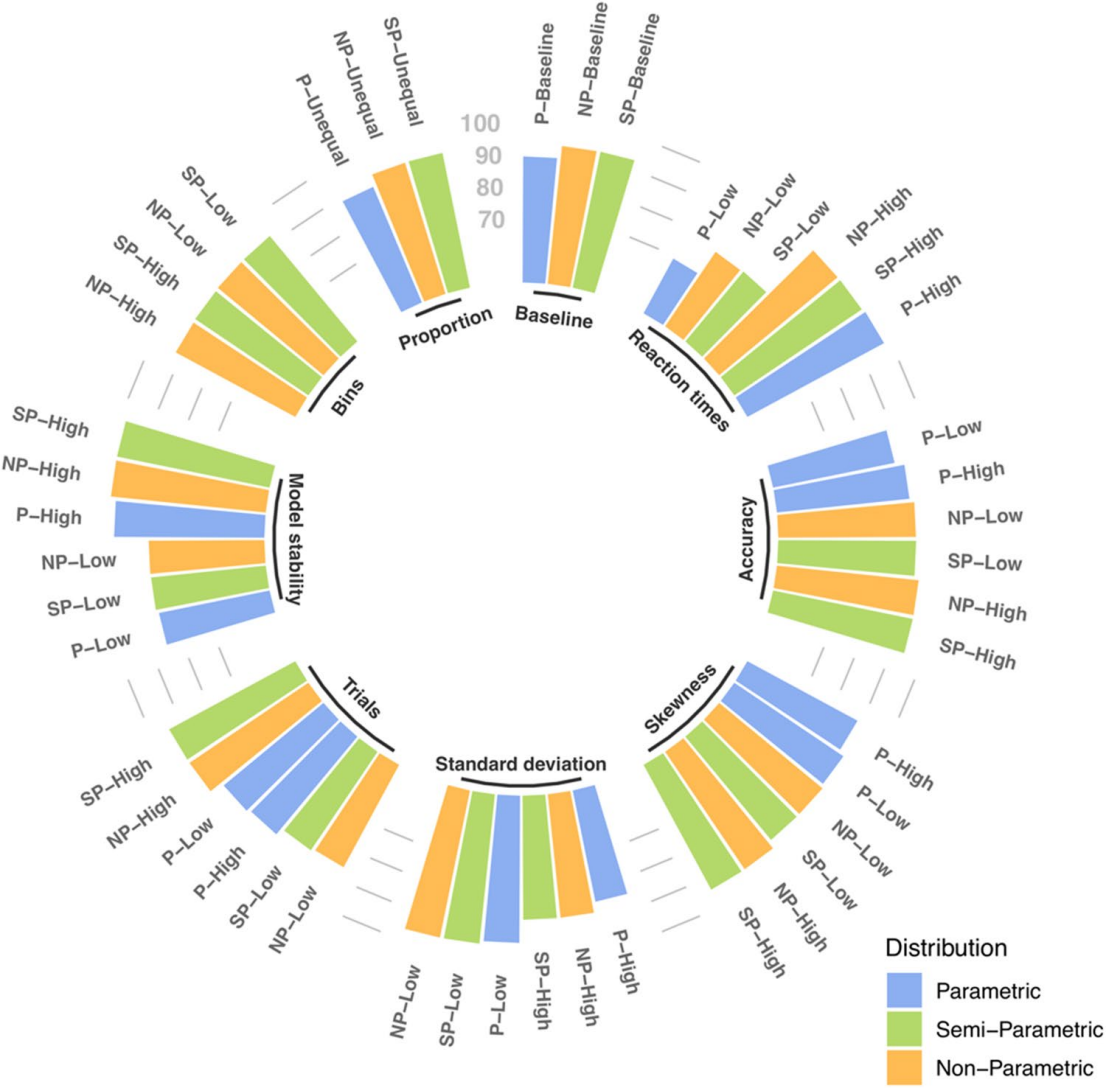
Model accuracy for Simulation 1. Within each manipulated feature, the different levels of non-parametric, semi-parametric, and parametric distributions are ordered according to model accuracy

In a first step, we analyzed how each mixture distribution correctly predicts the simulated hidden states with the baseline values. To achieve this, we computed, by distribution, the proportion of correctly classified trials for every simulated participant. A trial is correctly classified if simulated trials of the first state are assigned to the fastest and less accurate model-based state and if simulated trials of the second state are assigned to the slowest and more accurate model-based state. As shown in Table 2 and Fig. 1, the mean model accuracy for semi-parametric mixture model (M = 93.25%, SD = 1.59) and non-parametric mixture model (M = 93.21%, SD = 1.62) were superior to the parametric mixture model (M = 89.31%, SD = 15.91). In order to investigate the potential discrepancies between model fittings more in depth, model accuracy difference between each pair of mixture models (i.e., non-parametric minus parametric, semi-parametric minus parametric and semi-parametric minus non-parametric) was computed for each simulated participant. Regarding the comparison between semi-parametric and non-parametric mixture models, the mean difference was almost null (M = 0.05, SD = 0.22, range – 0.80 to 1.00). This is explained by the fact that a large majority of simulated participants (83%) showed similar model accuracy with both mixture models. A small proportion of simulated participants (13%) had a slightly better model accuracy for the semi-parametric mixture model over the non-parametric mixture model whereas the remaining 4% showed the reverse. Logically, the difference between non-parametric and parametric mixture models and the difference between semi-parametric and parametric mixture models were highly similar. The mean difference between non-parametric and parametric mixture models was 3.90% (SD = 2.49, range – 0.8 to 11.2), indicating an advantage for the non-parametric distribution. A better model accuracy for non-parametric model was observed for 97% of the simulated participants and a better model accuracy for the parametric model was found for the remaining 3%. Correspondingly, the mean difference between semi-parametric and parametric mixture models was 3.95% (SD = 2.45, range – 0.8 to 11.2), indicating an advantage for the semi-parametric distribution. A better model accuracy for semi-parametric model was found for 98 % of the simulated participants and the remaining 2% show the reverse.

**Table 2:** Model accuracy for Simulation 1

	Parametric	Semi-parametric	Non-parametric
Baseline	89.31 (2.82)	93.25 (1.59)	93.21 (1.62)
Reaction times difference			
Low	69.41 (6.49)	78.33 (5.35)	78.05 (5.38)
High	98.36 (0.77)	97.91 (1.13)	97.88 (1.14)
Accuracy difference			
Low	88.59 (3.06)	92.91 (1.69)	92.84 (1.70)
High	91.46 (2.55)	94.37 (1.29)	94.35 (1.28)
Skewness			
Low	90.98 (2.31)	91.82 (1.84)	91.78 (1.86)
High	88.98 (2.37)	94.27 (1.56)	94.22 (1.57)
Standard deviation			
Low	95.93 (1.32)	96.82 (1.22)	96.83 (1.23)
High	85.41 (3.66)	88.70 (2.25)	88.68 (2.29)
Model stability			
Low	85.13 (2.43)	85.89 (5.88)	86.01 (5.92)
High	96.78 (1.32)	98.47 (0.78)	98.45 (0.80)
Number trials			
Low	90.36 (4.67)	87.09 (10.35)	86.34 (11.08)
High	89.54 (1.44)	94.65 (0.70)	94.65 (0.70)
Number bins			
Low	NA	92.27 (2.20)	92.27 (2.20)
High	NA	92.25 (1.80)	92.23 (1.90)
Mixture Proportion	89.10 (3.00)	92.90 (1.80)	92.80 (1.90)

Mean and standard deviation (in parentheses) of parametric, semi-parametric and non-parametric model accuracy for the baselines as well as the different levels of the manipulated features

Figure 1 displays the parametric, semi-parametric and non-parametric model accuracies for each manipulated feature of the data. Table 2 provides the corresponding mean model accuracy and SD. For the RT manipulation, low and high levels influenced in a similar way the three mixture models with low level (i.e., increasing the overlap between both distributions) showing a worse model accuracy than high level (i.e., decreasing the overlap between both distributions). In addition, we observed a more important impact of this manipulation on the parametric model than on semi-parametric and non-parametric models. In other words, the difference in model performance between the baseline and low/high levels is larger for the parametric mixture model than non-parametric and semi-parametric mixture models. For all distributions, the accuracy manipulation has a small influence on model accuracy. A large difference (high level) in accuracy between both states is associated with a better model accuracy than a small difference (low level). It could also be noted that, regardless of the level of manipulation, non-parametric and semi-parametric mixture models always provided a better model accuracy. The skewness manipulation did not influence similarly the mixture models. Parametric mixture model showed a better model accuracy for less skewed distributions (low level) and a worse model accuracy for more skewed distributions (high level). Surprisingly, the reverse is observed for semi-parametric and non-parametric mixture models. In addition, as for the accuracy manipulation, non-parametric and semi-parametric mixture models provided a better model accuracy, regardless of the level of manipulation. The manipulation of standard deviation similarly impacts model accuracy of the three mixture models (i.e., better model accuracy for low level of manipulation and worse model accuracy for high level of manipulation). For trial manipulation, the sample size did not have the same impact on the distributions. While parametric model did not seem to be influenced by the sample size, a drop in model accuracy was observed for semi-parametric and non-parametric mixture models when the number of trials decreases. Model stability manipulation impacted model accuracy in similar way for the three distributions (i.e., worse model performance for low level of manipulation and better performance for high level of manipulation). The number of bins, defined for the semi-parametric and non-parametric mixture models, did not change model accuracy. This means that, in an acceptable range, this arbitrary parameter did not seem to have an impact on the performance of semi-parametric and non-parametric mixture models. Finally, varying the mixture proportion did not influence model accuracy and this for all mixture models.

Tables 3 and 4 (panel A) report model selection results varying the number of states for each of the three models with both statistics of goodness-of-fit, namely BIC and AIC. Non-parametric and semi-parametric mixture models correctly identified two states model as the optimal model with both AIC and BIC. Model selection with BIC showed that the two-states model fitted best for 71% and 88% of the simulated participants using the semi-parametric and the non-parametric distributions, respectively. An underestimation of states (i.e., one state as winning model) was observed for the remaining simulated participants. Model selection with AIC showed that, for these two distributions, the model with two states provided the best fit for all simulated participants. Parametric mixture model showed an overestimation of the number of states with both statistics of goodness-of-fit. Using BIC, three states fitted best for 82% of the simulated participants whereas the two-states model was correctly identified as the best-fitting model for the remaining 18%. Using AIC, the four-states model was selected as the best-fitting model for 99% of the simulated participants. For one participant, the best-fitting model was the model including three states.

**Table 3:** Model selection procedure with Bayesian information criterion (BIC)

Distribution	Models (n best)				Best Model
	1 State	2 State	3 State	4 State	
*A) Stimulation 1*					
Parametric	0	18	82	0	3 States
Semi-parametric	29	71	0	0	2 States
Non-parametric	12	88	0	0	2 States
*B) Stimulation 2*					
Parametric	0	0	22	78	4 States
Non-parametric	0	0	100	0	3 States
*C) Stimulation 3*					
Parametric	0	0	35	65	4 States
Non-parametric	2	98	0	0	2 States
*D) Experiment 1*					
Parametric	0	1	30	25	3 States
Non-parametric	4	48	4	0	2 States
*E) Experiment 2*					
Parametric	0	0	1	11	4 States
Non-parametric	0	12	0	0	2 States

The models (*n* best) columns represent the number of simulated participants for which this was the best model by mixture distribution. The best model column depicts the winning model by distribution based on *n* best results. Model selection procedure for Simulation 1 is depicted in panel A, for Simulation 2 in panel B, for Simulation 3 in panel C, for Experiment 1 in panel D, for Experiment 2 in panel E.

**Table 4:** Model selection procedure with Akaike information criterion (AIC)

Distribution	Models (n best)				Best Model
	1 State	2 State	3 State	4 State	
*A) Stimulation 1*					
Parametric	0	0	1	99	4 States
Semi-parametric	0	100	0	0	2 States
Non-parametric	0	100	0	0	2 States
*B) Stimulation 2*					
Parametric	0	0	0	100	4 States
Non-parametric	0	0	100	0	3 States
*C) Stimulation 3*					
Parametric	0	0	0	100	4 States
Non-parametric	0	100	0	0	2 States
*D) Experiment 1*					
Parametric	0	0	0	56	4 States
Non-parametric	0	16	27	13	3 States
*E) Experiment 2*					
Parametric	0	0	0	12	4 States
Non-parametric	0	4	6	2	3 States

The models (*n* best) columns represent the number of simulated participants for which this was the best model by mixture distribution. The best model column depicts the winning model by distribution based on n best results. Model selection procedure for Simulation 1 is depicted in panel A, for Simulation 2 in panel B, for Simulation 3 in panel C, for Experiment 1 in panel D, for Experiment 2 in panel E

#### Discussion

The findings of Simulation 1 showed that semi-parametric and non-parametric mixture models are associated with similar patterns of performance for the baseline values as well as for the different levels of manipulated parameters. The potential difference between both methods was further investigated with the baseline values. For a large majority of participants, no difference in model accuracy was observed between semi-parametric and non-parametric models.

The comparison of semi-parametric and non-parametric models with the parametric model indicated a better model accuracy for semi-parametric and non-parametric mixture models in almost all investigated situations. The only exceptions were the high level of RT manipulation and the low level of trials manipulation. Furthermore, the different levels of each manipulated parameter had, in most cases, the same influence on the distributions. One exception was the trials manipulation where a poor sample size seems to have a negative impact on semi-parametric and non-parametric mixture models only. Another exception was the skewness manipulation where more skewed distributions were associated with worse performance for parametric mixture model and with better performance for semi-parametric and non-parametric mixture models.

Finally and crucially, model selection results indicated that non-parametric and semi-parametric distributions correctly estimate the number of simulated states for both model selection criterions. This was not the case for the parametric distribution which overestimates the number of states with both model selection criterions.

### Simulation 2: Is parametric mixture model efficient to recover three hidden states?

It is not uncommon to find more than two strategies used by participants in cognitive psychological experiments (see for instance, arithmetic problems solving; LeFevre et al., 1996). It is therefore important to demonstrate that the non-parametric mixture method was able to efficiently identify more than two hidden states. In Simulation 2, three states rather than two were simulated. As for Simulation 1, RT and accuracy for each states were respectively drawn from a SWD and from a binomial distribution.

#### Method

RT and accuracy data for the three states were respectively sampled from three SWDs and three binomial distributions. More precisely, two states were identical to the baseline parameters of the Simulation 1. That is, the first state was associated with the following parameters: *M*_*1*_= 600 ms, *SD*_*1*_ = 100, *SK*_1_ = 1.5, accuracy_1_ = 70%. The second state had the following parameters: *M*_*2*_ = 900ms, *SD*_*2*_ = 150, *SK*_2_ = 1.75, accuracy_2_ = 90%. To stick as close as possible to the baseline features of Simulation 1 (i.e., 300 ms RT difference and 20% of accuracy difference), the new third state was generated with the following parameters: *M*_3_= 300 ms, *SD*_*3*_ = 70, *SK*_3_ = 1.25, accuracy_3_ = 50%. This new state could correspond to a participant not doing the task properly and/or adopting a guessing strategy, resulting in fast responding at chance performance.

We simulated 750 observations per participant (100 in total) with an equal mixture proportion (i.e., 250 observations per state). The transition parameters of the Markov chain were 0.8, 0.1, and 0.1, meaning that the three states have a probability of 0.8 of staying in the same state and a probability of 0.1 of switching to one of the other states. For the semi-parametric and non-parametric mixture models, the number of bins was fixed to 20. Finally, the fitting and model selection procedures were set up in a similar way as Simulation 1.

#### Results

Because the comparison between semi-parametric and non-parametric mixture models led to the same conclusion as Simulation 1, namely a model with or without the ordinal information elicited similar accuracy performance and model selection results, we only reported non-parametric and parametric results. Semi-parametric results of this section as well as the ones concerning the last simulation and the two applications to real data are detailed in Supplementary Information.

The results showed that both mixture methods recovered the three hidden states with a high accuracy. A slight advantage for the non-parametric (M = 95.65%, SD = 0.99) over the parametric model (M = 94.15%, SD = 1.63) was observed. In order to better capture individual differences between both mixture models, we computed the model accuracy difference for each simulated participant (non-parametric model accuracy minus parametric model accuracy). The mean accuracy difference was 1.50% (SD = 1.67, range – 2.00 to 8.00). For 80% of the simulated participants, non-parametric mixture model provided a better model accuracy than parametric mixture model while 18% showed the reverse. No difference was observed for the remaining 2%.

As shown in Tables 3 and 4 (panel B) reporting the model selection results, non-parametric mixture model correctly identified the three-states model as the optimal model with both AIC and BIC. More precisely, model selection with BIC and AIC showed that the three-states model fitted best for all simulated participants. For the parametric mixture model, the four-states model is the winning model for 78% of the simulated participants. The correct model (i.e., three-states model) was only identified in 22 simulated participants. Using AIC, an overestimation of the number of states (i.e., four-states model) was found for all participants.

#### Discussion

The specific aim of this second simulation was to demonstrate that the non-parametric mixture method was also efficient in recovering more than two hidden states. Therefore, a third simulated state was added to the two states already generated in Simulation 1. Results showed that all mixture methods recovered with a high precision (around 95%) the three hidden states. Again, no difference was found between semi-parametric and non-parametric mixture models, with a large proportion of simulated participants providing the same model accuracy (see Supplementary Information for more details). Regarding the comparison between non-parametric and parametric mixture models, a small advantage was found for the non-parametric one. In addition, an important proportion of the simulated participants showed a better model accuracy for non-parametric mixture model than parametric mixture model. Importantly, the non-parametric mixture model was able to capture the correct number of hidden states whereas the parametric mixture model overestimated it. More precisely, the non-parametric mixture model identified three states for all simulated participants with both AIC and BIC criteria. In contrast, the parametric mixture model estimated four states for a large majority of participants with BIC criterion and for all participants with AIC criterion.

### Simulation 3: Data generated by a cognitive model

Simulation 1 and 2 addressed the general question of what information of the RT distribution was required to reliably recover hidden cognitive states. We concluded that a non-parametric mixture model which does not require the exact shape of the RT distribution, nor the ordinal information encoded in the ordering of RT, was a good approach to estimate hidden states. In Simulation, we aim at validating this result by generating data with a well-established cognitive model of binary choices, the DDM. This simulation supports our claim that HMM-modeling of cognitive psychological data can be achieved when considering the right level of abstraction for the RT distribution.

As most sequential sampling models, the DDM assumes that decisions are based on the gradual accumulation of evidence for the various response alternatives until a preset boundary is reached. At this point, a response is initiated (Ratcliff, 1978; Ratcliff & McKoon, 2008). DDM includes four key parameters. *Drift rate* is the average rate at which evidence is accumulated towards one of two boundaries. *Boundary separation* indicates the amount of information required before a decision is made. The *starting point* of accumulation reflects a bias for one response over another. Finally, *non-decision time* quantifies the duration of processes outside the decision process (e.g., Mulder, Van Maanen, & Forstmann, 2014; Ratcliff & McKoon, 2008). Sequential sampling models, including the DDM, have been applied to many decision-making paradigms in order to study cognitive processes driving observed behavior (e.g., Archambeau, Forstmann, Van Maanen, & Gevers, 2019, 2020; Donkin & Van Maanen, 2014; Ratcliff, 1978; Ratcliff, Thapar, & McKoon, 2001; Van Maanen et al., 2012). Here, using DDM allows us to generate behavioral data (RT and accuracy) representative of latent cognitive states. Instead of random and arbitrary parameter values, DDM parameters were set to the fitted values of a participant taken from Mulder and collaborators (2013). In their study, they applied DDM to auditory decision-making performance involving speed–accuracy trade-off manipulation (Mulder et al., 2013). We used the fitted parameter values of speed and accurate conditions to simulate mixture data representing the two latent cognitive states (i.e., the fastest/less accurate state and the slowest/more accurate state, respectively).

#### Method

The data were generated from the DDM using the “rtdists” R package (Singmann et al., 2016). The DDM parameter values of participant “WB” (auditory stimuli, 10% coherence) from Mulder et al., (2013) were used for the simulation. More precisely, one state was generated based on the DDM parameter values of the accuracy condition (i.e., drift rate = .081, boundary separation = .203, non-decision time = .203). The other state was generated according to the DDM parameter values of the speed condition (i.e., drift rate = .081, boundary separation = .081, non-decision time = .596). The starting point was fixed to half of the boundary separation and the diffusion constant was s = .1. For the other simulation characteristics, we took the baseline values used in Simulation 1. That is, we simulated 500 observations per participant (100 in total) with an equal mixture proportion (i.e., 250 observations per state). The transition parameters of the Markov chain were 0.8 and 0.2, meaning that both states have a probability of 0.8 of staying in the same state and a probability of 0.2 of switching to the other state. For the semi-parametric and non-parametric mixture models, the number of bins was fixed to 20. Finally, the fitting and model selection procedures were set up in a similar way as Simulation 1 and 2.

#### Results

First, we controlled that the speed and accuracy DDM parameters taken from Mulder et al. (2013), indeed generated a fast/less accurate state and a slow/more accurate state. Averaged over all simulated participants, the state generated with DDM parameters of the speed condition had a mean RT of 754.20 ms (SD = 7.53) and a mean accuracy of 66.12% (SD = 2.82). The state generated with DDM parameters of the accuracy condition had a mean RT of 1677.23 ms (SD = 40.65) and a mean accuracy of 83.97% (SD = 2.33). Therefore, the hidden states were associated with the expected behavioral responses.

Model accuracy results showed that both mixture methods provided a high accuracy in recovering hidden states, with a slight advantage for the non-parametric (M = 94.87%, SD = 1.51) over the parametric model (M = 93.93%, SD = 1.67). As for Simulations 1 & 2, the model accuracy difference (non-parametric model accuracy minus parametric model accuracy) was computed for each simulated participant. The mean accuracy difference was 0.94% (SD = 2.19, range – 4.60 to 6.20). For 66% of the simulated participants, non-parametric mixture model provided a better model accuracy than parametric mixture model while 31% showed the reverse. No difference was observed for the remaining 3%.

As shown in Tables 3 and 4 (panel C) describing the results of the model selection procedure, the non-parametric mixture model correctly identified the two-states model as the optimal model with both AIC and BIC. More precisely, model selection with BIC showed that a two-state model fitted best for 98% of the simulated participants. An underestimation of the number of states (i.e., one state as winning model) was observed for the remaining 2%. Model selection with AIC showed that the model with two states provided the best fit of all participants. For the parametric mixture model, two-states model was never estimated as the optimal model with both AIC and BIC. Using BIC, four states fitted best for 65% of the simulated participants whereas three states fitted best for the remaining 35%. Using AIC, the four-states model was selected as the best model for all participants.

#### Discussion

The results of this section showed that all mixture methods identified with a high accuracy (> 90%) the hidden states simulated by the cognitive model. A very small advantage was observed for the non-parametric mixture model over the parametric one. Most of the simulated participants showed a better model accuracy for non-parametric mixture model than parametric mixture model. Concerning the model selection procedure, non-parametric mixture model correctly estimated the number of simulated states for both model selection criterions (i.e., AIC and BIC). This was not the case for the parametric distribution which always overestimated the number of states present in the data set of each simulated participants. This represents a crucial advantage for non-parametric over parametric mixture models*.* Indeed, even though the parametric mixture model accurately predicts the states when the correct number of states are assumed, there is no way to correctly assess the number of states in an initial model selection procedure. Accordingly, accurate state recovery at a trial level become only meaningful when one has strong theoretical arguments for assuming two states. Overall, the findings of Simulation 3 are fully in line with Simulation 1 and 2, therefore reinforcing their validity.

## Application to real data

In this section, we demonstrate that the non-parametric mixture model can be used on different cognitive psychological data to efficiently disentangle underlying states. To this end, the method was applied to two data sets from different fields of psychology: perceptual decision-making under time pressure and economic decision-making. In both data sets, the experimental conditions are intended to elicit different cognitive strategies, making these experiments good candidates for the validation of state detection methods. Note however, that in practice the purpose of HMMs is to identify unknown states, and one would never identify known experimental conditions. However, for the purposes of validation this seems a reasonable approach.

### Experiment 1: Perceptual decision-making

In Experiment 1, we applied the mixture methods to data from Miletic & Van Maanen (2019; Experiment 2)^4^. The goal of their study was to investigate how timing ability affects decision-making under time pressure. Participants had to indicate which of the two displayed circles flashes most often (a so-called expanded judgment task; e.g., Brown, Steyvers, & Wagenmakers, 2009; Hawkins, Brown, Steyvers, & Wagenmakers, 2012; Katsimpokis, Hawkins, & Van Maanen, 2020; Smith & Vickers, 1989 ; Van Maanen, Fontanesi, Hawkins, & Forstmann, 2016). In a short deadline block, participants had to indicate their choice before an individually set deadline, inducing time pressure. In a long deadline block, the deadline was fixed to 5 s, ensuring no time pressure. The order of both blocks was counterbalanced across participants. The final sample size included 56 participants (for more details about the experimental procedure, see Miletic & Van Maanen, 2019). Here, we address the hypothesis that the behavior between the two blocks differs categorically, resulting in a two-state HMM as the optimal model. That is, time pressure induced in the short deadline block favors speed over accuracy, resulting in a faster but less accurate state than in the long deadline block.

The fitting and model selection procedures were similar to the simulation studies. The experimental conditions were considered as ground truth to assess model accuracy. Therefore, a trial is correctly classified if the model-based state assignment corresponds to the fastest model-based state in the short deadline block and to the slowest model-based state in the long deadline block.

#### Results

**Fig. 2:**
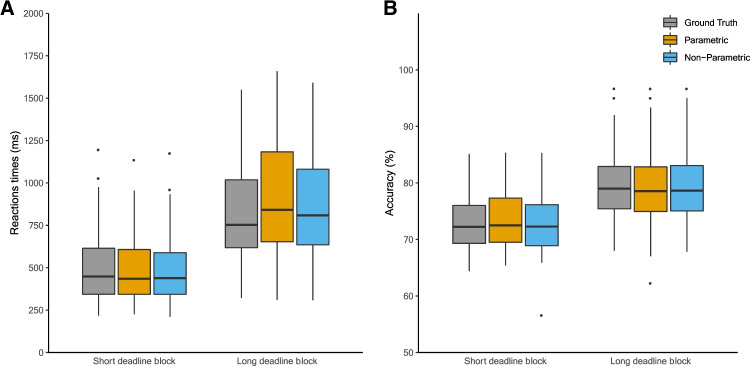
Behavioral characteristics of perceptual decision making data. Boxplot displaying RT and accuracy for the ground truth (experimental conditions) and model-based states for parametric and non-parametric distributions

In a first quality check, we analyzed whether the ground truth (i.e., experimental conditions) and the two mixture methods, non-parametric and parametric, have the expected behavioral responses. Compared to the long deadline block, the short deadline block was associated with faster RT (510 vs. 830 ms) and less accurate responses (73 vs. 80%). Both mixture models defined the behavioral characteristics of the states in agreement with the experimental conditions. That is, both mixture models estimated a relatively faster/less accurate state, which was associated with the short deadline block and a relatively slower/more accurate state which was associated with the short deadline block (see Fig. 2).

The mean model accuracies for parametric and non-parametric mixture models were 86.36% (SD = 12.34) and 88.41% (SD = 12.14) respectively. Again, the difference in model accuracy between both mixture models (i.e., non-parametric minus parametric) was computed for each participant. The mean difference between non-parametric and parametric mixture models was 2.05% (SD = 4.60), with a range from – 6.64 to 16.82 . For 30 of the 56 participants, the non-parametric mixture model provided a better model accuracy than the parametric mixture model. A better model accuracy for the parametric mixture model was observed for 17 participants while no difference was found for the nine remaining participants.

**Fig. 3:**
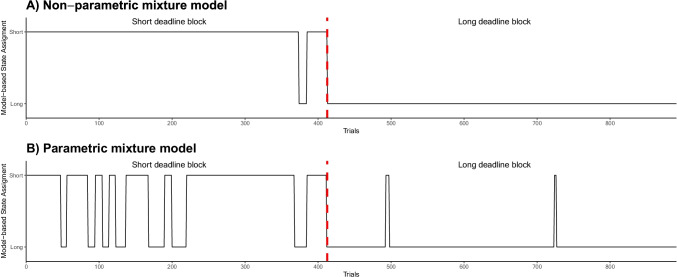
Time series of the model-based state assignment. Panel A displays the state assignment according to the non-parametric mixture model for one random participant. Panel B displays the state assignment over time according to the parametric fitting for the same panel participant. The *dashed red line* represents the switching from one experimental condition to another

Figure 3 shows the time series of the model-based state assignment for one selected participant according to the non-parametric fitting (panel A) and the parametric fitting (panel B). When the participant switched from one condition to another (represented by the dashed red line), we can see that both models are able to detect this strategy break in the data. That is, both models predicted a switch between states at this point in time. However, more strategy switches are observed in the model-based states of both non-parametric and parametric models, in particular in the short-deadline block. Under the assumption that this participant perfectly followed instructions, Fig. 3 reveals how the parametric model incorrectly assigns, more often than the non-parametric one, the slow state to trials belonging to the fast state. This explains why, for this specific participant, the strategy recovery was better for non-parametric than parametric model.

Regarding model selection with BIC, the two-states model was the best model for the non-parametric mixture model for a large majority of participants (≅ 85%). For the parametric mixture model, the three-states model fit best for a small majority of participants (54%, 30 participants) while the four-states model was the best model for almost all remaining participants (45%, 25 participants; see Table 3, panel D). Using AIC, the three-states model was selected as the best model for the non-parametric mixture model for ≅ 48% of participants (i.e., 27 participants). The remaining participants had two states (29%, 16 participants) or four states (23%, 13 participants) as the best model. Finally, the four-states model is the winning model of all participants for the parametric mixture model (Table 4, panel D). Under the assumption that participants follow instructions, this illustrates again how the parametric mixture model overestimates the number of cognitive strategies that are used.

### Experiment 2: Economic decision-making

In this section, we apply the non-parametric mixture model to disentangle strategies in economic decision-making such as choices between lotteries. This is an interesting case because research suggests that there are categorically different strategies for making these kinds of choices (Kahneman, Knetsch, & Thaler, 1991; McFadden, 1999; Rabin, 1998; Thaler, 1980). Lotteries are characterized by an integration of an amount that can be won, and a probability of winning that amount. Typically, the choice has to be made between a lottery with a low amount but a high probability of getting that amount versus a high amount but a low probability of getting it (e.g., 70% of chance of winning 10 euros vs. 30% of chance of winning 25 euros). Previous studies provided empirical support that choices are made depending on two different strategies: a value-based strategy and a default-option strategy (Guo, Trueblood,& Diederich, 2017; Kirchler et al., 2017; Kocher, Schindler, Trautmann, & Xu, 2019; Rubinstein, 2007; Couto et al., 2020). Under the value-based strategy, amount and probability are combined into a single value and the option associated with the highest value is selected. This strategy is rational (i.e., based on the highest expected outcome) but slow and effortful. However, the better choice in term of expected outcome is not constantly chosen. On the contrary, individuals are often biased towards a default-option such as a risk-averse choice (i.e., option with the highest probability). Compared to the value-based strategy, the default-option strategy is fast and not effortful (Rubinstein, 2007).

To demonstrate that the non-parametric mixture method correctly identifies both strategies, new data were collected where participants were instructed to use either the value-based strategy or the default-option strategy. The given instructions were then used as ground truth to assess model accuracy.

#### Method

Twelve participants (M = 22 years, SD = 5; ten women) took part in Experiment 2. Participants received either a course credit or a monetary reward (base amount of 8 euros) for their participation, with the possibility to get an extra amount up to 4€, depending on two randomly chosen trials (each trial corresponding to one instruction). The study protocol was approved by the local ethical committee of the University of Amsterdam. Informed consent was obtained by all participants included in the study. All participants were naïve with respect to the purpose of the experiment.

In the economic decision-making task, participants had to choose between two lotteries associated with different probabilities and amounts of potential gain. One lottery was characterized by a high probability (*p* > 50%; e.g., 80%) of winning a certain amount while the other had a smaller probability (100%-*p* ; e.g., 100-80 = 20%) of winning a higher amount. The lotteries were presented at the bottom-left and bottom-right of the screen. A graphical representation summarizing both lotteries was also used. Probabilities were shown by means of complementary areas of a circle, displayed on the middle of the screen. Amounts were represented by varying the height of a vertical bar. The vertical bar was displayed on the same side as the corresponding lottery (see supplementary Fig. 1). One lottery was associated with a higher expected value, with expected value computed as follows:

$$Expected\ value= Probability\times Amount$$

On half of the trials, the lottery with smaller probability/ higher amount had the highest expected value. On the other half, the lottery with higher probability/smaller amount had the highest expected value. Each trial started with a fixation cross (500 ms). Next, the two lotteries were displayed until a response was provided. The response was followed by a choice-confirmation screen (1000 ms), where a box was drawn around the selected lottery. Two experimental conditions involving different instructions were used: the Calculate condition and the Preference condition. These Calculate and Preference conditions refer to the value-based strategy and default-option strategy, respectively. In the Calculate condition, participants were instructed to choose the lottery with the highest expected value. An explanation about the expected value and how to compute it was given at the beginning of the experiment. In the Preference condition, participants were instructed to select the lottery according to their personal preference. There were five Calculate blocks and five Preference blocks of 64 trials each. The two conditions were presented alternatingly and the starting condition was counterbalanced across participants.

The fitting procedure was highly similar to the previous sections with the difference that the accuracy variable was replaced by a dichotomous variable indicating whether the lottery with the highest expected value was selected or not. As for accuracy, expected value was modelled as a multinomial distribution with an identity link function. The instructions (i.e., Calculate and Preference conditions) were considered as ground truth for model accuracy. We assume that the value-based strategy is characterized by slower and more rational (i.e., highest expected value) choices than the default-option strategy. Consequently, we consider a trial correctly classified if the model-based state assignment corresponds to the fastest model-based state in the Preference blocks and to the slowest model-based state in the Calculate blocks. No change was made concerning the model selection procedure.

#### Results

**Fig. 4:**
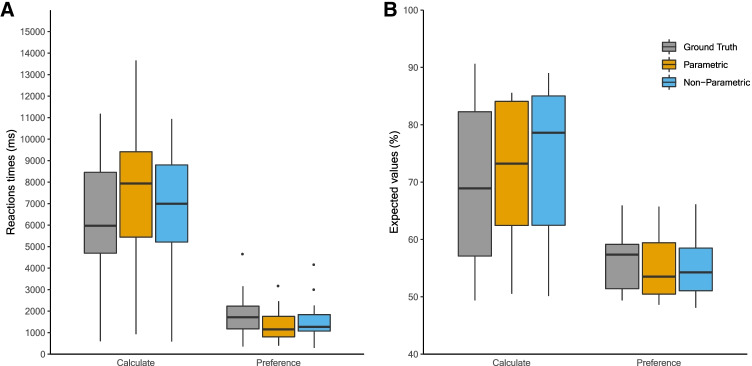
Behavioral characteristics of economic decision-making data. Boxplot displaying RT and accuracy for the ground truth (experimental conditions) and model-based states for parametric and non-parametric distributions

As expected, both instructions were associated with different behavioral characteristics. As shown in Fig. 4, participants were, on average, slower and selected more often the highest expected value in the Calculate than in the Preference block. Importantly, both parametric and non-parametric mixture models estimated two states with similar behavioral characteristics (i.e., faster/less expected value choices versus slower/more expected value choices) to the one of the instructions.

In agreement with previous results, the mean model accuracy for non-parametric model (M = 84.06%, SD = 15.72) was superior to the parametric model (M = 76.95%, SD = 15.91). The difference in model accuracy between both mixture models also provided a similar pattern of results as before. The mean difference between non-parametric and parametric was 7.11% (SD = 9.20), with a range from – 4.53 to 26.56. For ten of the 12 participants, non-parametric mixture model provided a better model accuracy than parametric mixture model while the two remaining participants showed the reverse.

Model selection with BIC showed that the two-states model is the best model for all participants with the non-parametric distribution. For the parametric model, the four-states model fit best for almost all participants (11 participants; Table 3, panel E). Using AIC, the three-states model is the best model for non-parametric distribution for 50% of participants (i.e., six participants). The remaining participants had two states (33%, four participants) or four states (17%, two participants) as best model. Finally, the four-states model is selected as the best model of all participants for the parametric distribution (Table 4, panel E).

### Discussion

The application of mixture methods on two different data sets revealed a highly consistent pattern of results. For both data sets, the methods identified states with behavioral characteristics similar to the experimental manipulation (i.e., the ground truth). Importantly, the non-parametric mixture model provided a better model accuracy than the parametric mixture model. This advantage in performance was more robust for the economic decision making data set than for the perceptual decision-making data set. Finally, the non-parametric mixture model correctly estimated the number of states with BIC. This was not the case for the parametric model which overestimated the number of hidden states. Taken together, these results imply that the non-parametric mixture model can be used as an efficient tool to disentangle hidden states/strategies in cognitive psychological data. Determining the number of hidden states/strategies, their behavioral characteristics and their identification at the trial level is possible with the proposed method. It is interesting to observe that not all participants seem to have the same number of states. This may reflect an overestimation of the number states, but it may also reflect that our approximation of a ground truth by way of the experimental manipulation is not entirely accurate. That is, some participants may adopt more strategies to respond in a particular condition, increasing the number of states that we find. A similar reasoning could be applied to strategy recovery. Indeed, a “wrong” strategy recovery could be due either to misclassification from the model or another strategy used by the participant (i.e., an incorrect ground truth for that trial).

## General discussion

The aim of the present paper was to provide a reliable method to identify hidden strategies in cognitive psychological data. To this end, we developed the non-parametric mixture model based on a HMM. More precisely, we used a model-free approach where a particular shape of RT distribution does not need to be assumed. This avoids potentially unreliable results when the incorrect RT base distribution is selected in the fitting process (Molenaar et al., 2018). After elaborating on the main findings of simulated and real data studies, we then provided some practical guidelines for researchers wishing to apply the non-parametric mixture model on their own data set.

In two simulation studies, we tested the proposed method on data simulated from a SWD for RT and binomial distribution for accuracy. More precisely, the first simulation aimed to investigate which kind of mixture data induce a poor model performance. We showed that non-parametric HMMs can reliably estimate underlying states in a wide range of behavioral patterns. The aim of the second simulation was to demonstrate that the proposed method is also efficient in identifying more than two hidden states. This is an important result because it shows how a non-parametric HMM naturally generalizes to more likely scenarios with multiple underlying cognitive strategies. In a third simulation study, the parametric mixture model was applied on RT and accuracy data generated by way of a cognitive model, the DDM. Results of all simulation studies showed that the non-parametric method identified, with a high accuracy, hidden cognitive strategies. Simulation 3 complements Simulations 1 and 2 by illustrating how the method fares with a more realistic data structure, including a dependency between RT and accuracy. Importantly, in all simulations, the current method provided a better model performance than a parametric approach assuming a Gaussian RT distribution. This implies that the shape of RT distribution is not necessary information to reliably recover hidden strategies. This conclusion is congruent with the study of Molenaar et al., (2018) implementing a semi-parametric approach in the psychometric domain (see also Molenaar et al., 2019). Crucially, our results go a step further showing that the ordinal information encoded in the ordering of RT is not essential either. Indeed, the comparison between semi-parametric and non-parametric mixture models elicited similar accuracy performance with identical model fitting for the large majority of simulated participants. Only implemented in the psychometric field, this also demonstrated that the semi-parametric mixture model can be considered as a valid approach when applied to typical cognitive psychological data.

In Simulation 1, we additionally varied parameter features of the generated data to explore which kind of mixture data is susceptible to exhibit poorer model performance. Over all manipulations, decreasing the mean RT difference between both strategies has the strongest impact on model performance for the three approaches. Given that this RT manipulation induces more overlap between the distributions representing both strategies, disentangling hidden strategies becomes more difficult. For the rest, the different levels of each manipulated parameter had, in most cases, no discernible effect on model accuracy. One exception was the skewness manipulation where more skewed distributions were associated with worse performance for parametric mixture model only. Because semi-parametric and non-parametric mixture models remain agnostic about a particular shape of distribution, they are consequentially not affected by a factor violating an assumed distribution. To put it simply, it is expected that a factor influencing the normality of the distribution has only an impact on the model performance assuming this normality. The other exception was the sample size manipulation. Decreasing the number of trials exclusively affected non-parametric and semi-parametric mixture models with a drop in model accuracy of approximately 6% compared to their respective baseline value. While it seems that the proposed method is more sensitive to the sample size, we argue that this effect could be reduced by adapting the number of bins. As shown in Simulation 1, the number of bins in an acceptable range did not seem to have an impact on the performance of semi-parametric and non-parametric mixture models. It is therefore possible that 20 bins is no longer an acceptable number of bins for this smaller sample size. To test this assumption, the Low level of trials manipulation was refitted with a smaller number of bins (i.e., 10 bins instead of 20). This time, the mean accuracy was 90.55 and 90.60% for the non-parametric and semi-parametric mixture models, respectively, reducing the gap with the baseline values at 2.5% and then, corroborating our hypothesis.

On top of investigating an accurate strategy recovery, we explored whether the proposed method could correctly determine the number of hidden strategies present in the data. Therefore, a model selection was performed where we varied the number of states from 1 to 4. The results of all simulation studies showed that only the non-parametric and semi-parametric mixture models accurately estimated the number of hidden states, regardless of the statistic for goodness-of-fit used (in the present paper, AIC and BIC). Critically, the parametric mixture model always overestimated the number of hidden states. This is line with previous findings of Molenaar et al., (2018), which demonstrated that the parametric approach tends to predict a mixture in the data when only one state was generated. It is important to note that, even though the parametric mixture model also recovered the hidden states with a high precision, this state classification would become meaningless because not supported by a model selection procedure.

Finally, the non-parametric mixture model was tested on two real data sets to demonstrate its applicability in different fields of cognitive psychology. First, we applied the non-parametric mixture method to data from Miletic & Van Maanen (2019) which investigated how timing ability affects perceptual decision-making under time pressure. Their task included two blocks in which participants had to make perceptual decisions. The blocks differed in the presence or absence of a response deadline. We hypothesized that the block with time pressure (i.e., a strict response deadline) favors speed over accuracy, resulting in a faster but less accurate strategy than in the block without time pressure. The second application concerned the economic decision making field. New data were collected where participants had to choose between either a lottery with a low amount but a high probability of getting that amount or a lottery with a high amount but a low probability of getting it (e.g., 70% of chance of winning 10 euros versus 30% of chance of winning 25 euros). Participants were instructed to alternatively apply one of the two different strategies identified in the literature (e.g., Kahneman et al., 1991; McFadden, 1999; Rabin, 1998, Thaler, 1980): the value-based strategy and the default-option strategy. In one condition, participants had to choose the lottery with the highest expected outcome, referring then to the value-based strategy. In another condition, participants were instructed to use the default-option strategy which means choosing the lottery according to their personal preference. We assumed that the value-based strategy was characterized by slower and more rational choices than the default-option strategy. For both data applications, we first ensured that the different conditions exhibited the expected behavioral responses. Moreover, we analyzed whether the mixture models defined similar behavioral characteristics for the inferred states. The results indicated that the observed behavior was coherent with both experimental manipulations and crucially, this was accurately captured by the different approaches. In a next step, the experimental manipulation was used as ground truth to assess model accuracy. As for simulation studies, non-parametric mixture model was superior to the parametric approach for both data sets. Finally, model selection results showed that the correct number of states was only determined by the non-parametric model and the semi-parametric model (see Supplementary Information) with BIC statistic for goodness-of-fit. In contrast, the parametric model again overestimated the number of hidden states present in the data. Taken together, the conclusions drawn in simulation studies hold in the context of real cognitive data sets, strengthening the validity of the non-parametric mixture model.

### Practical guidelines

In a general way, the developed method focused on one of the most often used variables in cognitive psychology: RT^5^. We approached the issue of inferring a hidden RT distribution by categorizing this variable into bins. Obviously, the method could be used with continuous variables other than RT as well. Moreover, the HMM approach allows to associate the categorized RT with other (multiple) variables apart from accuracy of the response (for a specific tutorial on HMM, see Visser 2011).

The major decision concerning the non-parametric mixture model is the number of bins which discretize the individual RT data. As previously demonstrated in Simulation 1, this number does not have an impact on model performance in an acceptable range number. The “ideal” number is intrinsically linked to number of trials present in the data. Based on our analyses, we suggest categorizing the RT data into bins in such a way that the ratio bins vs. total number of trials is approximately 0.05. In addition, the number of bins should be no less than 5 and no more than 35.

While our results did not show any potential effect from outliers in the data, we recommend being careful about extremely slow outliers. Imagine a scenario where the individual RT distribution includes one extremely slow outlier. The categorization of RT would lead to a last bin with one trial, a lot of empty middle bins (i.e., bins without any trial) and one or two first bins with all the remaining trials. In such an extreme configuration, the fitting of the non-parametric model would lead to unreliable results, since the effective number of bins drops below our recommendation of a minimum of five bins. In summary, we advise researchers to look to a good repartition of trials into the bins.

Thirdly, even though a specific set of cognitive strategies could be inferred from the literature, we suggest validating such a hypothesis by performing an initial model selection procedure at the individual level. Indeed, it is possible that a participant does not use all the set of the available strategies to perform a task (e.g., applying one strategy even when multiple strategies are possible; e.g., Lemaire & Arnaud, 2008). Consistent with previous applications of HMMs (e.g., Visser 2011) and in line with the results of this study, BIC should be preferred as a statistic for goodness of fit.

A final consideration concerns the variability of stimulus materials. To reduce the complexity of the models, we refrained from explicitly modeling the items. This choice seems warranted given the high predictive accuracy, also in the applications to real data sets. Nevertheless, it is imaginable that variation in stimulus materials covaries with state assignment. Taking this variation into account potentially improves the accuracy. An easy-to-use hierarchical non-parametric model that includes item effects would therefore be a natural and desirable extension of this work.

### Conclusions

In this paper, we proposed a new method – the non-parametric mixture model – to disentangle hidden strategies in cognitive psychological data. This method is based on an HMM fitting in which a particular shape of RT distribution does not need to be assumed. This has a considerable advantage given that a wrong inference about the base distribution could lead to unreliable results (Molenaar et al., 2018). Through simulation studies and applications on real data, we repeatedly demonstrated that this method is more efficient than a parametric approach which typically tends to overestimate the number of states. The non-parametric mixture model allows researchers to accurately determine the number of hidden strategies present in the data, their behavioral characteristics and their identification at the trial level. It is therefore a useful statistical tool that can be applied in many areas of cognitive psychology.

## Footnotes

1 Therefore, throughout this paper, the terms “state” and “strategy” are used interchangeably to refer to a set of processes that entail a specific pattern of behavioral responses.

2 In many case, the RTs are log-transformed to better conform to the normal distribution.

3 Supplementary information is made available online (https://osf.io/87qju)

4 The perceptual decision-making task from Experiment 1 was not selected because we assumed only one processing state for this experiment given the extremely simple experimental design.

5 It should be noted that if the “true” distribution for the underlying state is known beforehand (e.g., from previous studies in the same experimental context), we advise the researcher to use this “true” distribution as fitting for RTs.
